# HDAC-dependent decrease in histone crotonylation during DNA damage

**DOI:** 10.1093/jmcb/mjz019

**Published:** 2019-03-13

**Authors:** Enas R Abu-Zhayia, Feras E Machour, Nabieh Ayoub

**Affiliations:** Department of Biology, Technion–Israel Institute of Technology, Haifa, Israel

**Dear Editor,**

The DNA damage response (DDR) ensures repair of DNA lesions caused by endogenous and exogenous mutagens that constantly threaten genomic integrity. Defective DDR results in accumulation of DNA lesions that could potentially lead to genomic instability and tumorigenesis. Posttranslational modifications (PTMs) including phosphorylation, methylation, acetylation, ubiquitination, and sumoylation play a central role in sensing, signaling, and repairing damaged DNA. Currently, it remains unknown whether lysine crotonylation (Kcr) is implicated in DDR. Kcr is an evolutionary conserved and abundant PTM that occurs in all core histones, and it promotes gene expression, at least in part, by providing binding sites to Yaf9, ENL, AFL, Taf14, Sas5 (YEATS) domain- containing proteins (Tan et al., 2011; Li et al., 2016). In addition, it was shown that hundreds of nonhistone proteins implicated in various biological processes, such as RNA processing and gene expression, undergo crotonylation at their lysine residues (Xu et al., 2017). Similar to acetylation, Kcr is a reversible modification that is catalyzed by the activity of p300 `writer’ protein and removed predominantly by class I histone deacetylases (HDACs) ‘eraser’ proteins. Furthermore, it was reported that sirtuin family deacetylases (SIRT1-3) exhibit modest decrotonylase activity (Wei et al., 2017).

Since gene expression is switched off at DNA damage sites (Polo, 2017; Abu-Zhayia et al., 2018) and given the emerging role of Kcr in regulating gene expression, we sought to determine Kcr levels upon DNA damage induction. Toward this end, U2OS cells were subjected to laser microirradiation and costained for γH2AX and pan crotonylated lysine antibodies. Results show local reduction in pan Kcr at laser-microirradiated sites marked by γH2AX (Figure 1A). Notably, the reduction in Kcr level is transient. Maximum decrease is observed at 5 min postirradiation, after which the amount of Kcr is gradually restored to basal level at 1 h after irradiation (Supplementary Figure S1). Next, we wanted to determine whether histone crotonylation is reduced at DNA damage sites. To do so, we monitored the levels of a specific crotonylated histone residue, H3K9 (H3K9cr). Our results show similar reduction in H3K9cr at laser microirradiated regions (Figure 1B), and hence we decided to focus on the changes in H3K9cr in the subsequent analysis.

Laser microirradiation induces complex DNA lesions (Aleksandrov et al., 2018). We tested therefore the levels of H3K9cr following the induction of different types of DNA damage. U2OS cells were exposed to ionizing radiation (IR), ultraviolet radiation (UV), or treated with etoposide (VP16) damaging agents, and the levels of H3K9cr were determined by western blot analysis. Results show rapid decrease in H3K9cr levels following IR, UV, and VP16 treatment (Figure 1C–E). Similar to laser microirradiation, the reduction in H3K9cr following DNA damage inflicted by IR, VP16, and UV is transient. Interestingly, while H3K9cr level is restored to basal level at 4 h after recovery from VP16 treatment (Figure 1E), the recovery time of H3K9cr after IR and UV is 12 and 24 h, respectively (Figure 1C and D). Unlike IR and VP16 treatments, we did not observe full recovery of H3K9cr level following UV radiation. These results suggest that the recovery (rate and extent) of H3K9cr after DNA damage is influenced by the type of genotoxic agents.

In agreement with a previous report (Wei et al., 2017), treating cells with SIRT-specific inhibitor, nicotinamide (NAM), results in a mild increase in Kcr, while HDACs inhibition using a specific inhibitor trichostatin A (TSA) leads to a severe increase in the levels of H3K9cr, suggesting that HDACs are the major lysine decrotonylases (Figure 1F). To determine which lysine decrotonylase mediates the reduction in H3K9cr after DNA damage induction, U2OS cells were treated with NAM inhibitor prior to VP16 treatment. Results show comparable reduction in Kcr after DNA damage in mock and NAM-treated cells (Figure 1G). On the other hand, TSA treatment suppresses the reduction in H3K9cr after either VP16 treatment or UV radiation (Figure 1H and I). Altogether, we concluded that the decrotonylase activity of HDACs, but not sirtuin, fosters the DNA damage-induced reduction in Kcr.

Previously, it was shown that HDACs accumulate at DNA damage sites and promote histone deacetylation (Miller et al., 2010). Our data suggest that HDACs have a dual role in regulating both the levels of lysine acetylation (Kac) and Kcr at DNA breakage sites. We speculate that the simultaneous DNA damage-induced reduction in Kac and Kcr contribute to gene silencing at DNA damage sites.

**Figure 1:**
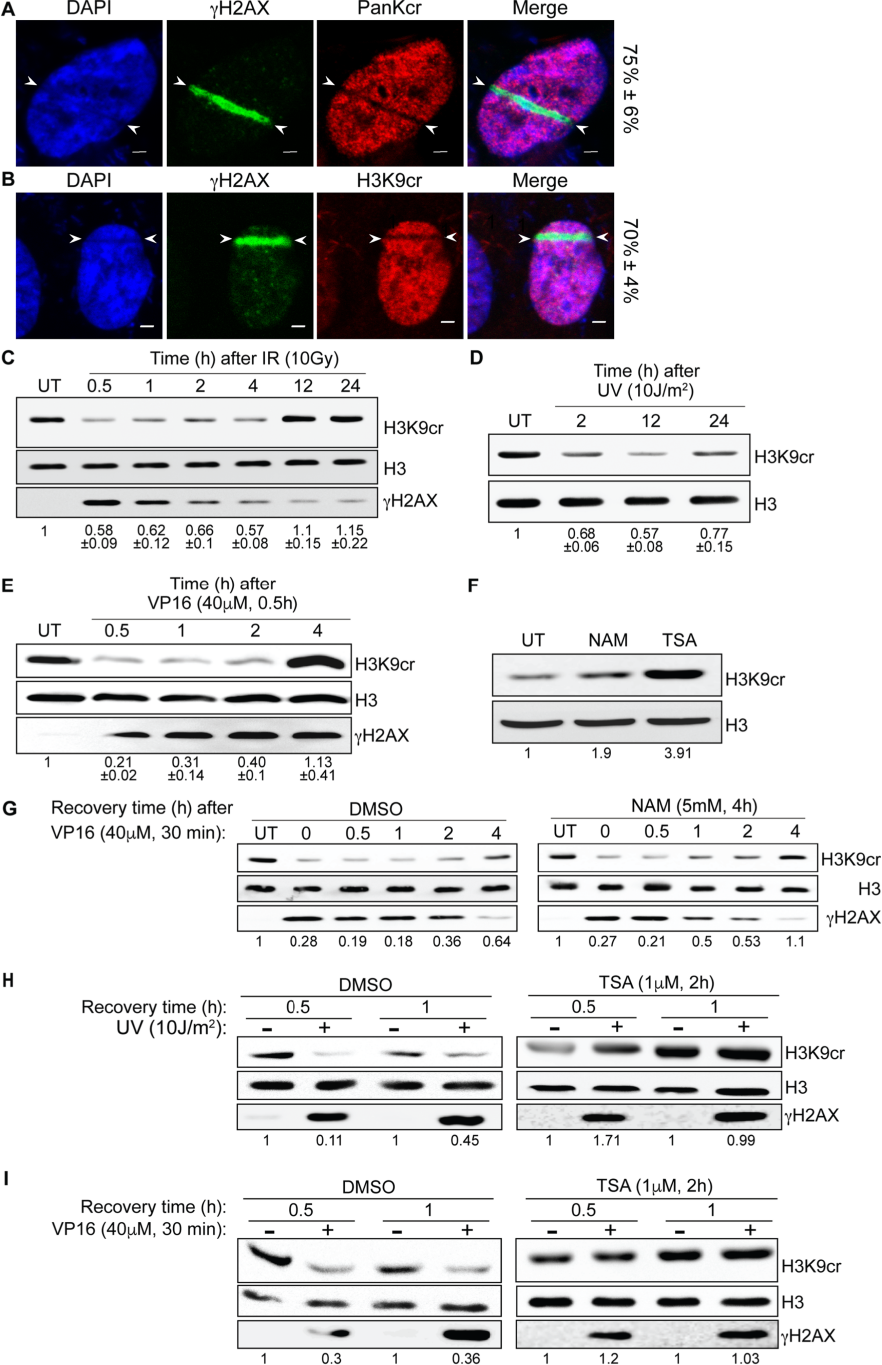
Kcr is reduced at DNA damage sites in an HDAC-dependent manner. (**A** and **B**) Representative images of U2OS cells that were exposed to laser microirradiation and 5 min later fixed and costained using the following antibodies: pan crotonyllysine (PTM-BIO; PTM-501, Zhejiang, China), crotonylated H3K9 (PTM-BIO; PTM-516), and γH2AX (Cell Signaling; 2577, Danvers, MA, USA). DNA was stained with DAPI (blue). Results are typical of four independent experiments (*n* > 50). The percentage of cells showing colocalization of the indicated markers is written on the right. Scale bar is equal to 2 μm. (**C**–**E**) show H3K9cr levels at different time points after DNA damage induced by IR (10 Gys; C), UV (10 J/m^2^; D), and VP16 (40 μm; 30 min; E). Histones were prepared by acidic extraction and subjected to western blot analysis. Histone H3 (Abcam; ab1791, Cambridge, MA, USA) is used as a loading control. γH2AX is used as a marker for DNA damage induction. The numbers below the blots indicate the ratio between the intensities of H3K9cr and total H3 bands, which was normalized to the untreated samples and averaged from three independent experiments. Band quantification was performed using ImageJ software. (**F**) Western blot shows the levels of H3K9cr after treatment with 5 mM NAM for 4 h (Sigma; N0636, Rehovot, Israel) or 1 μm TSA (Sigma; T1952) for 2 h. (**G**) as in E except for pretreating the cells either with DMSO or NAM prior to VP16 treatment. (**H** and **I**) as in D and E except for pretreating the cells either with DMSO or TSA prior to DNA damage induction. The two antibodies used in this study, pan crotonyllysine (PTM-501) and crotonylated H3K9 (PTM-516), have been tested for their selectivity and specificity by at least two independent groups (Tan et al., 2011; Andrews et al., 2016). Bands quantification was performed as described above.

Interestingly, DNA damage induction is accompanied by dynamic changes, consisting of increase and decrease, in the levels of H3K9 trimethylation (Ayrapetov et al., 2014). We hypothesize that the reduction in Kcr might be prerequisite for the alteration in H3K9 methylation levels during DNA damage. Since the same catalytic domain of HDACs regulates Kac and Kcr, it is unfeasible to decipher the alleged crosstalk between Kcr and lysine methylation. Identifying a mutant or an inhibitor that selectively targets the decrotonylase, but not the deacetylase, activity of HDACs will be highly beneficial for studying the interdependence between Kcr and lysine methylation in DDR.

Altogether, our data implicate for the first time Kcr in DDR and describe a hitherto unrecognized role of HDACs in counteracting histone crotonylation during DNA damage. This intriguing discovery, linking histone crotonylation to DDR, provides opportunities for novel therapeutic interventions and raises many open questions related to the biological function of histone crotonylation and its interplay with other histone modifications at DNA damage sites. In this regard, alterations in other crotonylated lysines of histone and nonhistone proteins during DDR are awaiting to be discovered.

## Supplementary Material

JMCB-2018-0507_Supplementary_material_mjz019
