# Supplementary material for: HDAC-dependent decrease in histone crotonylation during DNA damage
Source: J Mol Cell Biol. 2019 Mar 13;11(9):804–6. doi: 10.1093/jmcb/mjz019 (PMC6821229; doi:10.1093/jmcb/mjz019)
Supplement: JMCB-2018-0507_Supplementary_material_mjz019 [file jmcb-2018-0507_supplementary_material_mjz019.pdf]

Supplementary material

HDAC-dependent decrease in histone crotonylation during DNA damage

Enas R. Abu-Zhayia*, Feras E. Machour*, and Nabieh Ayoub[1]

* These authors contributed equally to this work
Department of Biology, Technion - Israel Institute of Technology, Haifa 3200003, Israel.

[1]Corresponding author: Prof. Nabieh Ayoub, Faculty of Biology
Technion - Israel Institute of Technology
Haifa 3200003, Israel.
Tel: +972-4-8294232 Fax: +972-4-8225153
Email: ayoubn@technion.ac.il

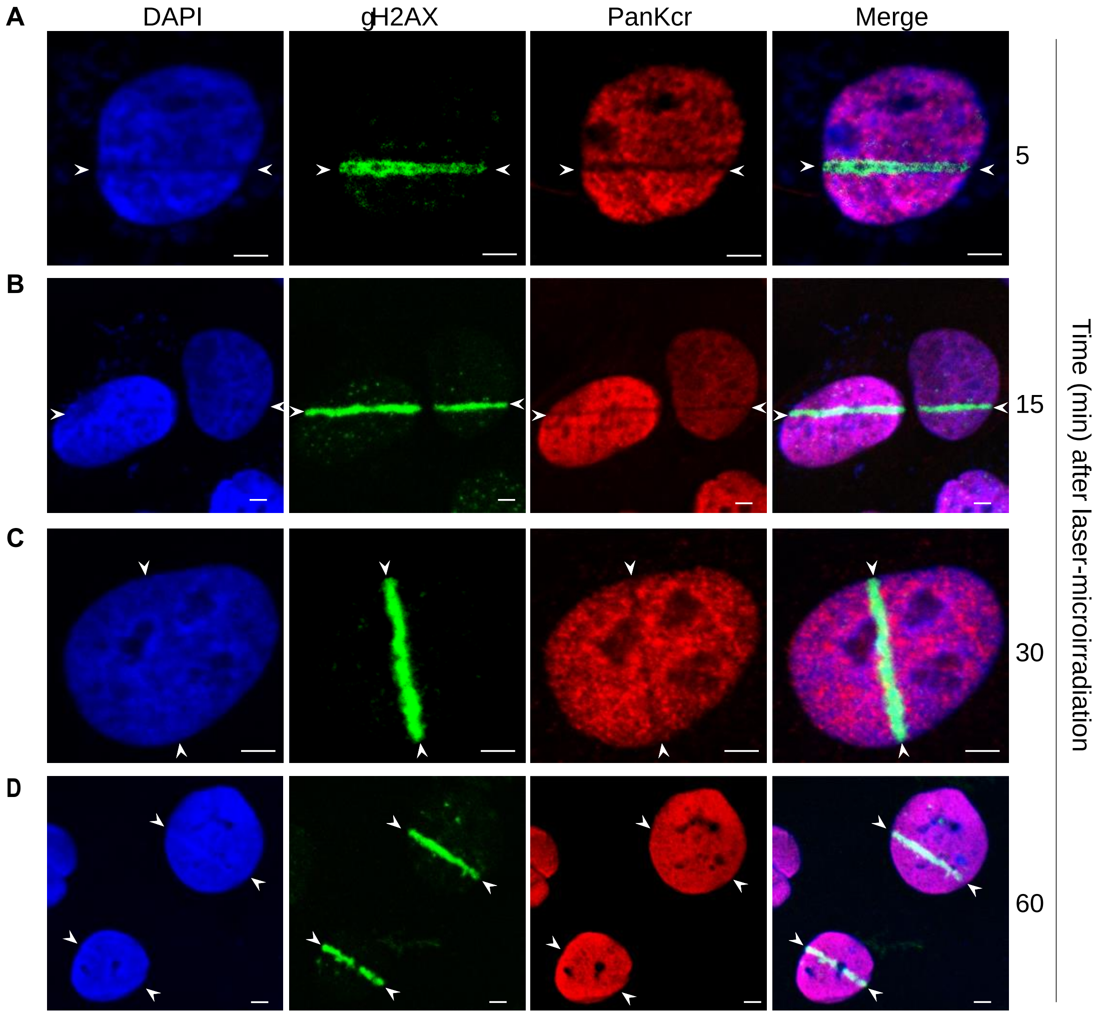

Supplementary Figure S1. Rapid and transient decrease in lysine crotonylation at laser-microirradiated sites. (A-D) Representative images of U2OS cells that were subjected to laser-microirradiation and costained with γH2AX and Pan crotonyllysine (PTM-BIO, PTM-501) antibodies at the indicated time points after irradiation. DNA was stained with Hoechst (blue). Results show that the reduction in the intensity of the Kcr signal at DNA damage sites peaks at 5 minutes post irradiation, followed by gradual recovery to basal level at 1 hour after damage induction. Scale bar, 2 µm.
